# Vaccination Knowledge, Attitudes, Perceptions, and Educational Needs of Pharmacists in Singapore: A Cross-Sectional Study

**DOI:** 10.3390/vaccines12111219

**Published:** 2024-10-26

**Authors:** Ally L. Au, Deborah M. Chia, Pei-Shi Ong

**Affiliations:** 1 Department of Pharmacy and Pharmaceutical Sciences, Faculty of Science, National University of Singapore, Singapore 117543, Singapore; ally_li_ling_au@nuhs.edu.sg; 2 Department of Pharmacy, Alexandra Hospital, Singapore 159964, Singapore; 3 Department of Pharmacy, National University Hospital, Singapore 119085, Singapore

**Keywords:** vaccine provider, pharmacists, attitudes, knowledge, educational needs

## Abstract

Background: Singapore’s adult vaccination coverage is suboptimal, and this can be attributed to a lack of vaccination recommendations and misconceptions. Studies have explored pharmacists’ vaccination knowledge, attitudes, and practice behaviour overseas but limited information about pharmacists in Singapore is available. This study aims to investigate pharmacists’ vaccination knowledge, attitudes towards providing vaccination services, and their educational needs. Methods: A cross-sectional study was conducted on pharmacists in various practice settings using an anonymous online survey. Results: Pharmacists’ vaccination knowledge (median: 2; IQR: 1–3), confidence in providing vaccination services (median: 6; IQR: 5–8), and frequency of providing vaccination services (median: 2; IQR: 1–3) were only average. However, 74.3% of pharmacists agreed that they play an important role as vaccine advocates. Apparent differences in knowledge level (*p* = 0.008), confidence level (*p* = 0.001), attitude (*p* < 0.001), and the frequency of educating patients (*p* = 0.001) and recommending vaccinations (*p* < 0.001) were observed among pharmacists from different practice settings. The main barriers identified were discomfort with giving injections (38.5%) and a lack of skills or knowledge (28.3%) at the point of survey. Conclusions: Pharmacists see the importance of their role as vaccine advocates. However, their vaccination knowledge, confidence in providing vaccination services, and practice behaviours are only average due to inadequate training. Continuous professional education is required to increase pharmacists’ readiness to provide vaccination services.

## 1. Introduction

Vaccination is a cost-effective strategy that can greatly reduce incidences of vaccine-preventable diseases (VPDs) such as measles and polio and prevents deaths [1]. In Singapore, the successful implementation of the National Childhood Immunisation Programme, which covers vaccinations against several VPDs, including tuberculosis and hepatitis B, has led to high vaccination coverage and low incidences of those diseases [2,3]. However, vaccination coverage among adults for VPDs, such as influenza and pneumococcal disease, have consistently been reported to be suboptimal, despite the initiation of the National Adult Immunisation Schedule [4,5,6]. The reasons for low vaccination uptake by adults in Singapore include misconceptions about vaccinations, insufficient opportunities for vaccination, and the lack of recommendation by healthcare professionals (HCPs) [4,7].

Currently in Singapore, pharmacists, doctors, and nurses serve as vaccine advocates who promote and recommend vaccines for patients. However, vaccinations are largely provided by doctors and nurses. Several studies have shown an increase in vaccination rates in countries such as Canada and the United States after pharmacists were also allowed to administer vaccinations [8,9,10,11]. Common reasons cited for the increase in vaccination uptake include increased convenience and access, and shorter waiting times [10,11,12,13]. Goad et al. showed that among patients younger than 65 years who were vaccinated at community pharmacies, 39% received vaccinations during off-clinic hours, and more than one million vaccinations were given during lunch hours, signifying the accessibility that pharmacies provide for the working population [11]. This suggests that pharmacists, especially community pharmacists (CPs), can reach out to the healthy adult population. This is also the demographic group largely neglected by doctors and nurses as they do not routinely visit clinics and hospitals. Patients were also found to be satisfied with having pharmacists as vaccine providers [12,13,14]. Pharmacists have been shown to play a significant role during pandemics such as the 2009 H1N1 pandemic and the COVID-19 pandemic, where they were authorised to administer vaccines, thus helping to achieve widespread vaccination coverage rapidly [15,16,17,18,19].

Little is known about the vaccination knowledge, attitudes, perceptions, and educational needs of pharmacists in Singapore. This information is required to identify possible knowledge gaps and areas for improvement in terms of attitudes and practice behaviours, to provide insights into designing educational programmes, and to propose appropriate interventions to enhance the role of pharmacists in vaccination services. This can enhance pharmacists’ role as vaccine advocates, and potentially as vaccine providers, thereby increasing Singapore’s vaccination uptake. Additionally, overseas studies mainly discussed the role of community pharmacists in providing vaccination services and did not evaluate the role of hospital pharmacists (HPs). 

Therefore, the objective of this study is to assess pharmacists’ vaccination knowledge, to determine their attitudes and perceptions towards providing vaccination services, to identify their perceived barriers as potential vaccine providers, and to identify their educational needs.

## 2. Materials and Methods

A cross-sectional study was conducted using an anonymous online survey. A pilot test was conducted by five practising pharmacists, including three Infectious Disease pharmacists, for relevance, accuracy, clarity of survey questions, and length of survey. The finalised survey was mounted online (Appendix A, Table A1) and disseminated to a list of approved pharmacies listed on the website of the Singapore Pharmacy Council (SPC). The SPC is the authority that governs and regulates the professional conduct and ethics of registered pharmacists in Singapore. Within this capacity, the SPC also maintains the register of pharmacists in Singapore. At present, this register contains 3989 pharmacists with 87.6% of them in active practice. Among those in active practice, 55.4% of them work in the public sectors in pharmacies within the hospitals and polyclinics as well as within various government-related agencies such as the Ministry of Health. The remaining 44.6% work in the private sector such as in various pharmaceutical companies or community pharmacies. The list of approved pharmacies from the SPC comprises pharmacies within the hospitals, the polyclinics, and the community that are directly serving patients. Polyclinics in Singapore are government-owned one-stop healthcare centres providing subsidised primary care. Within these centres, pharmacists mainly work alongside family physicians to care for patients with chronic diseases through the provision of specialised pharmacist-run ambulatory care clinics in hypertension, hyperlipidemia, and diabetes as well as in medication management services. On the other hand, community pharmacies in Singapore are private-owned retail pharmacy chain stores where pharmacists mainly provide consultation and advice for minor ailment management. Following the dissemination of the survey, it was active for five weeks, from 19 August 2020 to 25 September 2020, where participation from pharmacists was entirely voluntary. The exclusion criteria were pharmacists in inactive practice, pharmacists not practising in direct patient care settings, and pharmacists who were pilot testers. 

The survey consisted of 21 questions covering four domains: (1) demographic information, including personal vaccination status, practice setting, and the duration of practice; (2) vaccination knowledge, which covered participation in vaccination educational programmes, information sources, and questions to assess vaccination knowledge; (3) attitudes and perceptions towards vaccination, which covered opinions on the role of pharmacists as vaccine advocates, confidence in providing vaccination services, practice behaviours, willingness to act as potential vaccine providers, and perceived barriers to pharmacists as vaccine providers; and (4) educational needs, including areas of interest in vaccination and the preferred type of educational programmes. Six questions regarding vaccinations were used to assess the participants’ knowledge, where they had to choose between “True”, “False”, and “Not sure” to best answer the questions. Five-point Likert scales that ranged from strongly agree to strongly disagree were used to assess the level of agreement for attitudes and perceptions. Questions on confidence in providing vaccination services had the options “Very confident”, “Confident”, “Moderately confident”, “Slightly confident”, and “Not confident”, while questions on the frequency of providing vaccination services had the options “Always”, “Often”, “Sometimes”, “Rarely”, and “Never”.

All statistical analyses were performed using SPSS software (Version 26). Survey data were analysed using descriptive statistics to summarise participants’ demographics and the frequency of responses. The Shapiro–Wilk test and Kolmogorov–Smirnov test were conducted to determine if variables were normally distributed. Likert scale items assessing attitudes and perceptions were dichotomised to “agree” (including “strongly agree” and “agree”) and “disagree” (including “neutral”, “disagree”, and “strongly disagree”). Data were analysed using a chi-square test, a Mann–Whitney U test, or a Kruskal–Wallis test where indicated. All analyses used two-tailed tests and statistical significance was accepted at *p* < 0.05.

The study was approved by the Department Ethics Review Committee (DERC) of the Department of Pharmacy and Pharmaceutical Sciences, National University of Singapore (PHA-DERC-06). Before participating in the study, participants were asked to provide informed consent by checking off their approval prior to the start of the survey. They were provided with comprehensive information about the study’s purpose and were assured that all information provided would be treated with strict confidentiality.

## 3. Results

### 3.1. Pharmacists’ Participation in Survey

A total of 400 survey responses were collected with 286 fully completed responses. There were 44 blank responses (11.0%) in which none of the survey questions were answered. There was a drop-out rate of 19.7%, as 70 responses were partially completed. Three pharmacists were not actively practising in direct patient care and their responses were removed from analysis. Data analysis was performed on completed responses only (*N* = 283) (Appendix A, Table A2).

### 3.2. Demographics of Pharmacists

The demographic characteristics of the respondents are described in detail in Table 1. Most respondents were female (70.3%), were within the age group of 20–29 years old, and were of Chinese ethnicity. More than half of the pharmacists received at least four vaccinations (76.3%) and the Measles–Mumps–Rubella vaccine was the most common vaccination received, with the exception of HPs, where the influenza vaccine was the most common vaccination received. The most common qualification among pharmacists was a bachelor’s degree in Science/Pharmacy. This is the basic degree for entry-level pharmacy practice in Singapore following the successful completion of pre-registration training and the passing of the national competency examination for entry-level practice. Following this, pharmacists in practice may upgrade their clinical skills with more advanced studies via the Master’s in Pharmacy and the Doctor of Pharmacy (PharmD) degrees. 

The majority of pharmacists were practising in the hospitals (63.6%), followed by the community pharmacies (26.5%) and the polyclinics (9.9%). While most of the pharmacists (76.9%) did not run specialised clinics, more than half of the polyclinic pharmacists (PPs) (60.7%) were running specialised clinics. The median number of years in practice among the respondents was 1–5 years. A large proportion of them (86.6%) did not participate in educational programmes regarding vaccinations. 

### 3.3. Pharmacists’ Vaccination Knowledge

**Figure 1:**
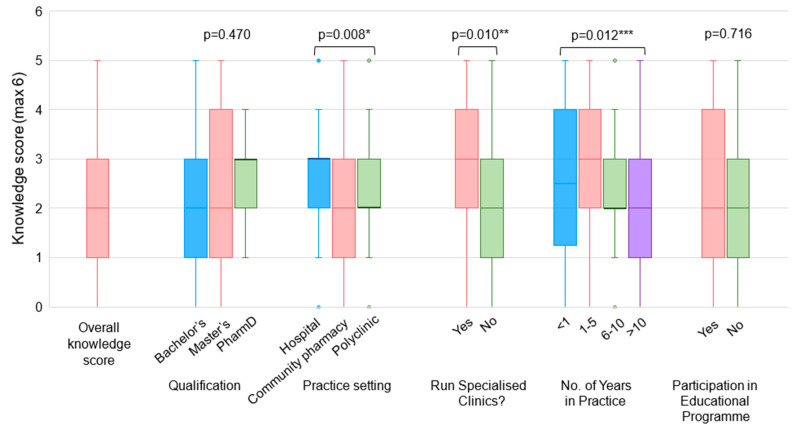
Pharmacists’ vaccination knowledge scores. * Statistically significant at *p* = 0.008 using Kruskal–Wallis test. Post hoc analysis identified statistically significant difference in knowledge scores between HPs and CPs. ** Statistically significant at *p* = 0.010 using Mann–Whitney U test. *** Statistically significant at *p* = 0.012 using Kruskal–Wallis test. Post hoc analysis identified statistically significant difference in knowledge scores between pharmacists with 1–5 years and >10 years of practice experience.

The median knowledge score of pharmacists was 2 (IQR: 1–3) (Figure 1). Vaccination knowledge on the simultaneous administration of vaccines and the angle of intramuscular administration were high at 54.4% and 74.5%, respectively, while knowledge on symptoms associated with anaphylaxis such as a request for water or thirst was the lowest at 13.8% (Appendix A, Table A2). Differences in knowledge scores were statistically significant based on the practice setting (*p* = 0.008) and the number of years in practice (*p* = 0.012). HPs had higher scores than CPs, and pharmacists who had practised for 1–5 years had higher scores than those who had practised for more than 10 years. Pharmacists running specialised clinics had higher vaccination knowledge than pharmacists who do not run specialised clinics (*p* = 0.010).

### 3.4. Confidence in and Perception of Pharmacist’s Role in Providing Vaccination Services

**Figure 2:**
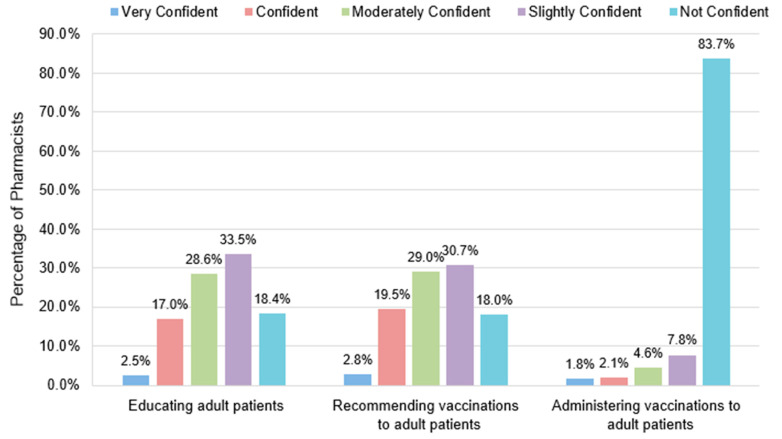
Pharmacists’ confidence in providing vaccination services. Very confident: I have the skills and knowledge to provide the service for all adult vaccines and I am able to train others to provide the service; confident: I have the skills and knowledge required to provide the service for all adult vaccines; moderately confident: I have adequate skills and knowledge required to provide the service in my current practice setting; slightly confident: I have some level of the skills and knowledge required to provide the service but am uncertain at times; not confident: I lack the skills and knowledge required to provide the service.

**Figure 3:**
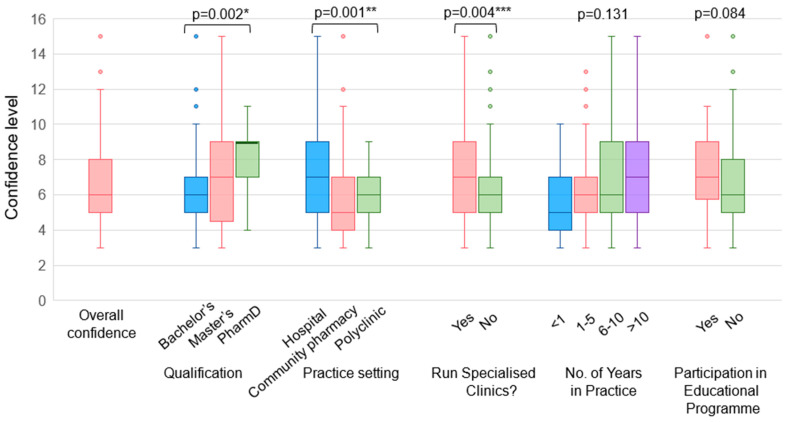
Overall confidence level of pharmacists in providing vaccination services. Confidence level (out of 15) is calculated as the sum of level of confidence in (1) educating patients about vaccinations, (2) recommending vaccinations, and (3) administering vaccines. A score of 5 (very confident): I have the skills and knowledge to provide the service for all adult vaccines and I am able to train others to provide the service; 4 (confident): I have the skills and knowledge required to provide the service for all adult vaccines; 3 (moderately confident): I have adequate skills and knowledge required to provide the service in my current practice setting; 2 (slightly confident): I have some level of the skills and knowledge required to provide the service but am uncertain at times; 1 (not confident): I lack the skills and knowledge required to provide the service. * Statistically significant at *p* = 0.002 using Kruskal–Wallis test. Post hoc analysis identified a statistically significant difference in confidence level between pharmacists with a bachelor’s degree and pharmacists with a PharmD degree. ** Statistically significant at *p* = 0.001 using Kruskal–Wallis test. Post hoc analysis identified a statistically significant difference in confidence level between HPs and CPs. *** Statistically significant at *p* = 0.004 using Mann–Whitney U test.

**Figure 4:**
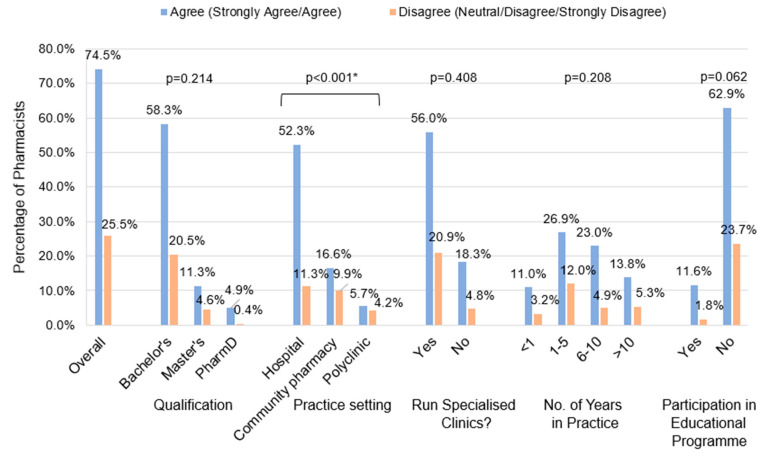
Pharmacists’ perception of the importance of their role in providing vaccination services. * Statistically significant at *p* < 0.001 using chi-square test. Post hoc analysis identified a statistically significant difference in the proportion of pharmacists who agree that they play an important role between HPs and CPs. Post hoc analysis also identified a significant difference in the proportion of pharmacists who agree that they play an important role between HPs and PPs.

Pharmacists had relatively low confidence in providing vaccination services, which includes educating patients about vaccinations, providing vaccination recommendations, and administering vaccinations to adult patients (Figure 2). A significant proportion of pharmacists responded that they do not have adequate skills and knowledge in educating patients on vaccinations (51.9%) and in recommending vaccinations (48.7%). In this survey, most pharmacists (83.7%) reported that they lack the skills and knowledge required to administer adult vaccinations. A statistically significant difference in confidence level was found among pharmacists with different qualifications (*p* = 0.002) and pharmacists from different practice settings (*p* = 0.001) (Figure 3). Pharmacists with a PharmD degree were more confident in providing vaccination services than pharmacists with a bachelor’s degree, and HPs were more confident than CPs. Pharmacists running specialised clinics were also more confident than those who do not run clinics (*p* = 0.004). Encouragingly, approximately three-quarters of the respondents (74.5%) agreed that pharmacists play an important role in the provision of vaccination services (Figure 4).

### 3.5. Practice Behaviour of Pharmacists

**Figure 5:**
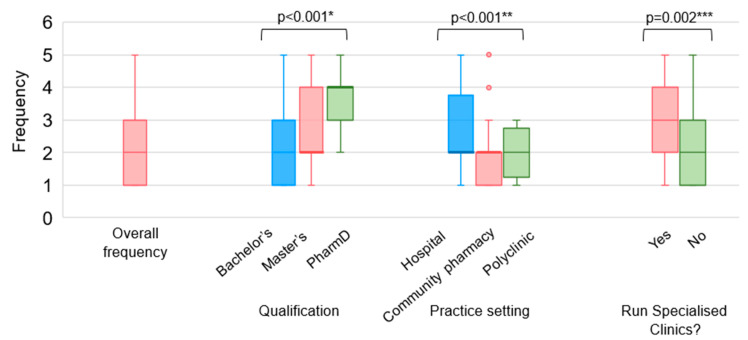
Practice behaviour of pharmacists. Frequency values: 5 (always): I provide the service at least once a week; 4 (often): I provide the service at least once a month; 3 (sometimes): I have provided the service at least once in the past 3 months; 2 (rarely): I have provided the service at least once during practice; 1 (never): I have never provided the service. * Statistically significant at *p* < 0.001 using Kruskal–Wallis test. Post hoc analysis identified a statistically significant difference in the frequency of providing vaccination recommendations between pharmacists with a bachelor’s degree and pharmacists with a PharmD degree. ** Statistically significant at *p* < 0.001 using Kruskal–Wallis test. Post hoc analysis identified a statistically significant difference in the frequency of educating patients between HPs and CPs. *** Statistically significant at *p* = 0.002 using Mann–Whitney U test.

Pharmacists reported a low frequency of providing vaccination services in the past three months with 67.9% and 62.6% of them not educating patients about vaccination and recommending vaccinations, respectively (Appendix A, Table A2). Pharmacists with a PharmD degree provided vaccination information to patients and recommended vaccinations more frequently than pharmacists with a bachelor’s degree (*p* < 0.05). HPs educated patients and recommended vaccinations more frequently than both CPs and PPs (*p* < 0.05). Additionally, pharmacists running specialised clinics educated patients and recommended vaccinations more frequently than pharmacists who do not run clinics (*p* < 0.05) (Figure 5).

### 3.6. Willingness to Serve as Vaccine Providers

At the point of survey, only 35.4% of pharmacists were supportive of pharmacist-led vaccination programmes (Appendix A, Table A2). No statistically significant associations were found between demographic characteristics and willingness to administer vaccinations. The most common reasons cited for willingness to be vaccine providers were that pharmacists have to have the capacity to provide the service if trained (n = 17), and that patients would benefit (n = 13) (Table 2). Among 88 pharmacists who explained why they were unwilling to administer vaccinations (neutral, disagree, and strongly disagree), the most common reasons cited were the availability of other HCPs to vaccinate (n = 25), the lack of confidence and familiarity with injections (n = 20), and the lack of training (n = 15). Regardless of pharmacists’ willingness to administer vaccinations, there was a common theme among pharmacists that CPs should be involved or should be prioritised to be vaccine providers.

### 3.7. Barriers to Pharmacist-Led Vaccination

In this study, key perceived barriers to implementing pharmacist-led vaccination in Singapore were identified. The top three barriers were being uncomfortable with injections (38.5%), a lack of skills or knowledge (28.3%), and a lack of time (14.5%) (Appendix A, Table A2). There were differences in the rankings based on the pharmacists’ practice setting. While the top three barriers for HPs were being uncomfortable with injections, a lack of skills or knowledge, and a lack of time, the top three barriers for CPs were being uncomfortable with injections, a lack of skills or knowledge, and a lack of space. PPs perceived a lack of skills or knowledge to be a larger barrier as compared to being uncomfortable with injections, but a lack of time was similarly the third barrier identified. Other barriers identified include a lack of professional indemnity and malpractice insurance for pharmacists, the safety of administering vaccines in community pharmacies, competition with current vaccine providers, and a lack of consolidated patient health records.

## 4. Discussion

This is the first study in Singapore to assess the knowledge, attitudes, and behaviours of pharmacists as vaccine advocates and their receptiveness towards the implementation of pharmacist-led vaccination programmes locally. The results revealed that pharmacists’ vaccination knowledge, confidence in providing vaccination services, and practice behaviours were only average and could be improved. Pharmacists in general perceived themselves to play an important role in providing vaccination services although most lacked confidence to be vaccine providers. 

Vaccination knowledge among pharmacists could be improved with median knowledge scores of 2 (IQR: 1–3). Similar findings of inadequate influenza vaccination knowledge among HCPs in Singapore, which include doctors and nurses, have also been reported by Sundaram et al. [20]. While Sundaram et al. reported inadequate influenza vaccination knowledge among HCPs, this current study suggests that pharmacists also had inadequate knowledge about other common vaccinations, such as the pneumococcal vaccination. Other studies also reported inadequate vaccination knowledge among pharmacists where the mean percentage knowledge score was around 50% [21,22,23]. However, due to differences in the study methodology and the difficulty of questions greatly differing across different studies, the findings may not be directly comparable. Differing knowledge results based on pharmacists’ practice setting and years of experience were apparent, with HPs having higher vaccination knowledge than CPs and pharmacists with 1–5 years of working experience having higher knowledge scores than those with more than 10 years of experience. Similarly, other studies on the knowledge of vaccination among HCPs revealed fewer years of working experience as a predictor of higher vaccination knowledge [21,24,25]. In Singapore, such a difference in vaccination knowledge could be because the content on adult national immunisation was taught within a pharmacy elective course that was not read by the majority of undergraduate students before 2020. Following a change in curriculum from the Bachelor of Science (Pharmacy) to the Bachelor of Pharmacy curriculum from 2020, childhood, adult, and travel vaccinations have been made compulsory for all students. The latter will thus better prepare future pharmacy graduates as vaccine providers. Meanwhile, the findings from this study thus highlight the need for practising pharmacists who did not have the chance to receive education on adult vaccinations previously to attain and increase their competency in this area through the attendance of Continuing Professional Education (CPE) programmes related to vaccinations. Such educational programmes would need to be of a certain rigour and should contain adequate content, both in theory and in the practice of vaccination, to effectively increase pharmacists’ vaccination knowledge and skills in vaccination. An example of such a programme is the Pharmacy-Based Immunization Delivery Certificate Training Program offered at the National University of Singapore, Department of Pharmacy and Pharmaceutical Sciences, in partnership with the American Pharmacists Association. 

It is notable that this study demonstrated positive overall attitudes toward vaccinations with approximately three-quarters of the respondents (74.5%) agreeing that pharmacists play an important role in providing vaccination services. A positive correlation between pharmacists’ attitude towards their role in providing vaccination services and practice behaviours is supported by previous studies [22,24]. It is also essential to build pharmacists’ confidence in providing vaccination services, especially in the local setting where currently there may be insufficient opportunities for CPs to be actively involved in vaccination services such as vaccine recommendations. In contrast to CPs, HPs and PPs, especially those who run specialised clinics, may have more opportunities to counsel patients about vaccinations and assess their need for vaccinations. They may have more frequent encounters with patients with chronic diseases such as heart diseases or immunocompromised patients who are the target population to receive vaccinations. On the other hand, CPs are in consistent contact with both the healthy population and outpatients. CPs can assist in overcoming barriers to vaccination by offering convenient access points, building confidence in vaccination, and actively increasing public awareness through health promotion programmes [26]. By involving pharmacists to actively educate on vaccination and recommend vaccinations, it can significantly increase vaccination uptake [8]. The relatively low confidence among survey respondents as vaccine providers suggests that to effectively encourage pharmacists to play a more active role in vaccination services, it may also be necessary to boost pharmacists’ confidence apart from merely augmenting their knowledge and skills in vaccination.

Perceived barriers towards the implementation of pharmacist-led vaccination programmes in Singapore should be considered when planning for pharmacists to be vaccine advocates and providers. Discomfort with handling injections and a lack of skills or knowledge were identified as the main barriers to implementing pharmacist-led vaccination programmes, which is consistent with other studies [23,27,28,29]. These two barriers can be addressed by providing adequate training programmes [8,10,30] through different modalities, with many respondents (87.3%) (Appendix A, Table A2) being interested in vaccination-related educational programmes delivered via in-person hands-on workshops (most preferred), online programmes, and in-person educational programmes. As survey respondents concurred that CPs should be prioritised to be vaccine providers likely due to the presence of other HCPs being available to administer vaccinations in hospital and polyclinic settings, barriers reported by CPs should be addressed to ensure the successful execution of pharmacist-led vaccination services. CPs reported a lack of space as one of the top three barriers, which was also highlighted by other studies [12,22,28,29]. Currently, most community pharmacies lack a designated area that can be used to administer vaccinations. This is consistent with the reasons stated by CPs for being unwilling to administer vaccines, such as a lack of confidentiality [31,32], hygiene concerns, and a lack of emergency preparedness to handle anaphylaxis [31]. Another study revealed that the Canadian public and HCPs were concerned with the capability of CPs in managing adverse reactions to vaccinations and the public perceived doctors and nurses to be better at managing adverse reactions [33]. CPs should thus be trained in this aspect to ensure they have the required competency to handle the acute events associated with vaccinations. 

In this study, although most pharmacists (64.6%) were not supportive of the expansion of their own scope of practice to include the administration of vaccines at the time of survey, the majority perceived themselves to play an important role in providing vaccination services. This finding is not surprising as pharmacists are not traditionally trained in vaccine administration in Singapore nor are involved in the administration of vaccines or other injectables routinely. Nonetheless, during the COVID-19 pandemic, dental assistants, pharmacists, and other allied health professionals in Singapore, who signed up under the Singapore Healthcare Corps as vaccinators, were successfully trained and given permission to administer vaccines. Their participation greatly helped reduce the workload of physicians and nurses, who are traditional vaccine administrators in Singapore, thereby easing the strain within the healthcare system. This example clearly exemplified the untapped potential of pharmacists in Singapore, particularly the CPs, to play a greater role as both vaccine providers and advocates, following appropriate training and certification. Such practice should be continued even as COVID-19 has now become endemic as Singapore is facing a rapidly aging population and has an ever-increasing healthcare expenditure. By allowing CPs as vaccine providers as long as they receive proper training and certification, they can aid in boosting the vaccination rate among healthy, older adults, thereby assisting in reducing the rates of VPDs. This will in turn help reduce healthcare expenditure associated with the treatment of VPDs and also preserve acute healthcare resources for sicker patients who are more in need of them. 

This finding was similarly reported in Hungary, Canada, and Thailand, where CPs were also not supportive of implementing pharmacist-led vaccination programmes [23,27,34] as pharmacists are not traditionally trained to administer vaccinations. Nonetheless, the concept of expanding pharmacists’ involvement in vaccination began in 1993 and is not new [35]. Numerous countries overseas have demonstrated the successful implementation of pharmacist-led programmes, with a slow but steady increase in demand for pharmacist-led vaccination after its implementation [8,10,36,37]. Lum et al. reported increased vaccination rates when involving pharmacists in the Asia Pacific [38]. This finding, together with pharmacists’ enthusiasm to be trained in immunisation advocacy and delivery as elucidated by this study, highlights that pharmacists can be part of the global effort against VPDs but also assist in the identification of those who qualify to receive vaccinations. An example will be elderly patients who are candidates for the Shingles vaccine. This can potentially aid in the prevention of Shingles-related complications, which is again particularly relevant in a rapidly aging society like Singapore. Additionally, following the proper upskilling of vaccination knowledge, pharmacists are ideally positioned to provide advice on the feasibility of simultaneous administration of multiple vaccines, which could boost vaccine uptake rates, and to be involved in the monitoring of patients’ immunisation records to ensure proper vaccine dosing based on recommended intervals. In particular, in Singapore, pharmacists could check to ensure that patients’ vaccinations are updated in the National Electronic Health Record (NEHR) System in a timely manner and to ensure that patients have their follow-up vaccination appointments scheduled with reminders sent to patients via the HealthHub app (a national digital healthcare companion app for Singapore citizens and permanent residents to access their health records and manage their medical appointments). With this, pharmacists serve to ensure that patients complete their vaccination series in a timely manner. 

In Singapore, under the Collaborative Practitioners Prescribing Programme (CP3), pharmacists or advanced practice nurses have been allowed to prescribe under a collaborative practice agreement with a medical practitioner. This allows pharmacists or advanced practice nurses to play a more active role in taking care of stable patients, thereby freeing physicians’ time to take care of sicker patients. This in turn facilitates the better use of healthcare resources and manpower albeit with an overlap of professional responsibilities. In a similar manner, allowing CPs as vaccine administrators in addition to traditional vaccine administrators such as nurses and physicians helps complement each other’s efforts in keeping the nation healthy despite overlapping professional responsibilities. Such a model is feasible by fostering a culture of mutual respect, clear communication, and collaboration as with the CP3 programme. It can in turn lead to a greater achievement of the Ministry of Health’s goal of supporting residents in leading healthier lifestyles through preventive care, regular health screening, and appropriate vaccinations. 

Lastly, a suitable and cost-effective pharmacist-led vaccination programme with pharmacists given access for updating patients’ vaccination records in the NEHR and ensuring that patients’ vaccination appointments are accurately scheduled should be piloted in Singapore. This can be carried out following further studies to evaluate existing models overseas to determine the most feasible model for this initiative in Singapore. 

There are some limitations to this study. Although the number of participants is high, it may not be sufficient to reflect pharmacists’ beliefs and attitudes across the country. Limited survey questions (six questions) were used to assess pharmacists’ vaccination knowledge. This small number of questions may be insufficient to accurately determine the knowledge gaps of pharmacists and further studies may be required. The sample size of HPs, CPs, and PPs were uneven, where the majority (63.6%) were HPs. Thus, the responses may be biased to the views of HPs. However, this study still provides valuable insights into the vaccination knowledge level, attitudes, and practice behaviour of pharmacists in Singapore.

## 5. Conclusions

Pharmacists generally see the importance of their role as vaccine advocates. However, their average knowledge and lack of confidence as vaccines providers, in part due to a lack of vaccination-related training and education, can be improved through continuous education and training tailored to their needs. This will help to better equip them to lead in community pharmacist-led vaccination programmes which can contribute to the nation’s goal of supporting residents in leading healthier lifestyles through preventive care, regular health screening, and appropriate vaccinations.

## Figures and Tables

**Table 1:** Demographics of participating pharmacists.

Characteristics	All Pharmacists *N* = 283 *N (%)*	Hospital Pharmacists (HPs) *N* = 180 *N (%)*	Community Pharmacists (CPs) *N* = 75 *N (%)*	Polyclinic Pharmacists (PPs) *N* = 28 *N (%)*
**Gender**				
Male	84 (29.7%)	54 (30.0%)	24 (32.0%)	6 (21.4%)
Female	199 (70.3%)	126 (70.0%)	51 (68.0%)	22 (78.6%)
**Age**				
20–29	143 (50.5%)	93 (51.6%)	35 (46.7%)	15 (53.6%)
30–39	110 (38.9%)	72 (40.0%)	27 (36.0%)	11 (39.3%)
40–49	22 (7.8%)	12 (6.7%)	8 (10.7%)	2 (7.1%)
50–59	6 (2.1%)	2 (1.1%)	4 (5.3%)	0 (0.0%)
≥60	2 (0.7%)	1 (0.6%)	1 (1.3%)	0 (0.0%)
**Race**				
Chinese	270 (95.4%)	175 (97.2%)	68 (90.6%)	27 (96.4%)
Malay	3 (1.1%)	1 (0.6%)	2 (2.7%)	0 (0.0%)
Indian	7 (2.5%)	4 (2.2%)	2 (2.7%)	1 (3.6%)
Others: 2 Vietnamese, 1 Filipino	3 (1.1%)	0 (0.0%)	3 (4.0%)	0 (0.0%)
**Number of vaccinations received (out of 5)**				
Received 0	2 (0.7%)	1 (0.6%)	1 (1.3%)	0 (0.0%)
Received 1	7 (2.5%)	3 (1.6%)	4 (5.3%)	0 (0.0%)
Received 2	18 (6.4%)	12 (6.7%)	6 (8.0%)	0 (0.0%)
Received 3	40 (14.1%)	18 (10.0%)	21 (28.0%)	1 (3.6%)
Received 4	102 (36.0%)	66 (36.7%)	26 (34.7%)	10 (35.7%)
Received 5	114 (40.3%)	80 (44.4%)	17 (22.7%)	17 (60.7%)
**Vaccinations received**				
Measles–Mumps– Rubella (MMR)	256 (90.5%)	161 (89.4%)	67 (89.3%)	28 (100.0%)
Hepatitis B	252 (89.0%)	161 (89.4%)	64 (85.3%)	27 (96.4%)
Tetanus, Diphtheria, & Acellular Pertussis (TdAP)	245 (86.6%)	157 (87.2%)	61 (81.3%)	27 (96.4%)
Influenza	244 (86.2%)	171 (95.0%)	46 (61.3%)	27 (96.4%)
Varicella	144 (50.9%)	95 (52.8%)	30 (40.0%)	19 (67.9%)
**Qualifications**				
Bachelor of Science/Pharmacy	223 (78.8%)	138 (76.7%)	62 (82.7%)	23 (82.1%)
Master’s	45 (15.9%)	28 (15.5%)	13 (17.3%)	4 (14.3%)
PharmD	15 (5.3%)	14 (7.8%)	0 (0.0%)	1 (3.6%)
**Status of Practising Certificate**				
Pre-registration/conditional registration	21 (7.4%)	19 (10.6%)	1 (1.3%)	1 (3.6%)
Active status	262 (92.6%)	161 (89.4%)	74 (98.7%)	27 (96.4%)
**Practice Setting**				
Hospital	180 (63.6%)	-	-	-
Community pharmacy	75 (26.5%)	-	-	-
Polyclinic	28 (9.9%)	-	-	-
Running any specialised clinics? *				
Yes	62 (23.1%)	22.8 (23.8%)	4 (5.9%)	17 (60.7%)
No	206 (76.9%)	72.8 (76.2%)	64 (94.1%)	11 (39.3%)
**Number of years in practice**				
<1 year	40 (14.1%)	31 (17.2%)	5 (6.7%)	4 (14.3%)
1–5 years	110 (38.9%)	63 (35.0%)	36 (48.0%)	11 (39.3%)
6–10 years	79 (27.9%)	53 (29.4%)	18 (24.0%)	8 (28.5%)
>10 years	54 (19.1%)	33 (18.4%)	16 (21.3%)	5 (17.9%)
**Participation in educational programme**				
Yes	38 (13.4%)	17 (9.4%)	15 (20.0%)	6 (21.4%)
No	245 (86.6%)	163 (90.6%)	60 (80.0%)	22 (78.6%)

* There were 15 blank responses for this question. The baseline varies from the total number of responses (n = 268, 172, 68, and 28 for all pharmacists, HPs, CPs, and PPs, respectively).

**Table 2:** Reasons cited by pharmacists for being willing and unwilling to serve as vaccine providers.

	Number of Responses	Quotes
List of themes for pharmacists who are willing to serve as vaccine advocates		
Pharmacists have the capacity to provide the vaccination service if trained - pharmacist-led vaccination services have been implemented overseas	17	“If we are capable of screening, we should be able to administer too. Pharmacists in other countries are trained to administer vaccine.” “As a(n) active team member of collaborative practice with Doctors, Pharmacists should be given the opportunity to learn how to administer vaccines.”
Beneficial to patients - convenience, shortened waiting time, providing one-stop vaccination services (counsel, recommend, administer)	13	“Avoid unnecessary visit to clinics which can free up slots for other patients.” “It minimizes the number of touch points for patient hence improving care and efficiency.”
List of themes for pharmacists who are unwilling to serve as vaccine advocates		
There are other healthcare professionals available to provide the service - structure of healthcare system	25	“No need to take on this role in the hospital setting when we have nurses able to do it. Even the doctors are not administering them.” “I think it is a good opportunity to learn something new, but I cannot see this service being time-efficient for pharmacist at the current moment (due to strong competition with other HCP for such service).”
Lack confidence and familiarity with handling injections	20	“Would need skills and practice before I am comfortable in doing it” “Not confident to do so due to lack of training and experience”
Lack of training and competency	15	“Lack of training. No competency in administration.” “Not trained in injection techniques”

## Data Availability

All research data can be found in Appendix A.

## Appendix A

**Table A1:** Survey questions distributed to participants.

Demographics	
1	Please indicate your gender: Male Female
2	Please indicate your age: 20–29 30–39 40–49 50–59 ≥60
3	Please indicate your race: Chinese Malay Indian Others
4	Please indicate the vaccinations that you have received (Please select all that apply): Influenza Measles-Mumps-Rubella (MMR) Varicella Tetanus, Diptheria & Acellular Pertussis (TdAP) None of the above
5	Please indicate your highest academic qualification: Bachelor of Science/Pharmacy Masters PharmD Others
6	Please indicate the status of your Practising Certificate: [SKIP TO Q9 if “Inactive status” is selected] Pre-registration/Conditional registration Active status Inactive
7	Please indicate your current practice setting: For locum pharmacists, please select others and indicate your practice setting Hospital Community Pharmacy Polyclinic Pharmacy Others
8	Are you currently running any clinics or specialised services? Yes No Not applicable
9	Please indicate your number of years in practice: <1 1–5 6–10 11–20 21–30 >30
**Knowledge**	
10	Have you participated in any educational programs (modules, workshops, etc.) regarding vaccinations? Yes No
11	Please select the information sources that you usually consult to obtain information about vaccination (Please select all that apply) Local vaccination guidelines (e.g.,: MOH, Society of Infectious Disease Singapore) International guidelines (e.g.,: CDC, APhA) Medical/Scientific resources (e.g.,: UpToDate, Micromedex, Mayo Clinic, scientific literature) Educational programs (e.g.,: workshops, courses) Mass media/Internet (e.g.,: personal blogs) Healthcare Professionals (e.g.,: pharmacists, doctors, nurses, etc.) Others
12	Please answer the following questions to the best of your ability.
12a	It is recommended to avoid giving live and inactivated vaccinations simultaneously. True False Not sure
12b	To administer an intramuscular injection, pinch the skin and insert the needle at a 45° angle. True False Not sure
12c	Apart from the usual symptoms of anaphylaxis, requests for water and thirst shortly after vaccination can hint that anaphylaxis is under way. True False Not sure
12d	The quadrivalent influenza vaccine covers against one influenza A H1N1 virus, one influenza A H7N9 virus and two influenza B viruses. True False Not sure
12e	For the hepatitis B vaccine, an interruption in the vaccination schedule (missed dose) does not call for extra doses or restarting the entire series of vaccination. True False Not sure
12f	The recommended vaccinations for patients <65 years old with chronic liver disease include the influenza vaccine and both pneumococcal vaccines (PCV13 and PPSV23). True False Not sure
**Attitude and Perception**	
13	I believe that Pharmacists have an important role to play in providing vaccination services. Strongly Agree Agree Neither Agree nor Disagree Disagree Strongly Disagree
14	Please rate your confidence level in providing the following vaccination services: *Very confident: I have the skills and knowledge to provide the service for all adult vaccines and I am able to train others to provide the service.* *Confident: I have the skills and knowledge required to provide the service for all adult vaccines.* *Moderately confident: I have adequate skills and knowledge required to provide the service in my current practice setting.* *Slightly confident: I have some level of the skills and knowledge required to provide the service but am uncertain at times.* *Not confident: I lack the skills and knowledge required to provide the service*.
14a	Educating patients about adult vaccinations Very confident Confident Moderately confident Slightly confident Not confident
14b	Recommending vaccinations for adult patients (directly to patients/through other healthcare professionals) Very confident Confident Moderately confident Slightly confident Not confident
14c	Administering vaccines to adult patients Very confident Confident Moderately confident Slightly confident Not confident
15	Please rate how frequent you provide the following vaccination services *Always: I provide the service at least once a week.* *Often: I provide the service at least once a month.* *Sometimes: I have provided the service at least once in the past 3 months.* *Rarely: I have provided the service at least once during practice.* *Never: I have never provided the service.*
15a	Educating patients about adult vaccinations Always Often Sometimes Rarely Never
15b	Recommending vaccination for adult patients (directly to patients/through other healthcare professionals) Always Often Sometimes Rarely Never
16	If given the opportunity, I would like to be able to provide direct administration of vaccines to patients. *Please elaborate on why you have chosen the option.* Strongly Agree Agree Neither Agree nor Disagree Disagree Strongly Disagree
17	Which adult vaccines do you think should be directly offered by Pharmacists? (Please select all that apply) Cholera Haemophilus influenzae Type B Hepatitis A Hepatitis B Influenza Japanese Encephalitis Measles-Mumps-Rubella Pneumococcal Polio Rabies Tetanus, Diphtheria & Acellular Pertussis Tetanus Post-Exposure Prophylaxis Typhoid Varicella Yellow fever Zoster Potential COVID-19 vaccine Others (please specify, e.g.,: none of the above, etc.): -___________
18	Please rank the top 3 potential barriers for Pharmacists to implement direct administration of vaccines. Uncomfortable with administering injections Lack of knowledge or skills Lack of time Lack of space for administration of vaccines and storage of vaccines Lack of support from patients to have pharmacists vaccinate Lack of support from other healthcare professionals (e.g.,: doctors, nurses, etc.) Others (please specify): ________________
**Knowledge**	
19	Are you interested in future education programs on vaccination? *[SKIP questions 20 and 21 if “No” was selected]* Yes No
20	What are some areas regarding vaccination that you are interested to find out more about? (Please select all that apply) Indication and contraindication of vaccines Dose of vaccine, number and frequency of doses Management of side effects and adverse reactions Administration techniques Patient counselling Others (please specify): ____________
21	Which of the following is your preferred type of education program on vaccination? (Please select all that apply) In-person courses Large group lecture/talks Small group seminar Hands-on workshop Self-paced online courses Web-lecture (webinar) Modules (series of modules videos and interactive activities) Reading materials Others (please specify): ___________

**Table A2:** Survey responses.

Characteristics	*N* = 283
Gender	
Male	199 (70.3%)
Female	84 (29.7%)
Age in years	
20–29	143 (50.5%)
30–39	110 (38.9%)
40–49	22 (7.8%)
50–59	6 (2.1%)
≥ 60	2 (0.7%)
Ethnicity	
Chinese	270 (95.4%)
Malay	3 (1.1%)
Indian	7 (2.5%)
Others—2 Vietnamese, 1 Filipino	3 (1.0%)
Vaccinations received	
Hepatitis B	252 (89.0%)
Influenza	244 (86.2%)
Measles-Mumps-Rubella (MMR)	256 (90.5%)
Varicella	144 (50.9%)
Tetanus, Diptheria & Acellular Pertussis (TdAP)	245 (86.6%)
Highest qualifications	
Bachelor of Science/Pharmacy	223 (78.8%)
Masters	45 (15.9%)
PharmD	15 (5.3%)
Status of Practising Certificate	
Pre-registration/Conditional registration	21 (7.4%)
Active status	262 (92.6%)
Inactive	0 (0.0%)
Practice setting	
Hospital	180 (63.6%)
Community Pharmacy	68 (24.0%)
Locum Community Pharmacy	7 (2.5%)
Polyclinic	28 (9.9%)
Others	0 (0.0%)
Running any specialised clinics? (*N* = 268)	
Yes	62 (23.1%)
No	206 (76.9%)
Number of years in practice	
<1	40 (14.1%)
1–5	110 (38.9%)
6–10	79 (27.9%)
11–20	40 (14.1%)
21–30	10 (3.5%)
>30	4 (1.4%)
Participation in education programme	
Yes	38 (13.4%)
No	245 (86.6%)
Sources of information	
Local vaccination guidelines	248 (87.6%)
International guidelines	220 (77.7%)
Medical/Scientific resources	176 (62.2%)
Educational programs	24 (8.5%)
Mass media/Internet	7 (2.5%)
Healthcare Professionals	126 (44.5%)
**Knowledge**	
It is recommended to avoid giving live and inactivated vaccinations simultaneously.	
True	65 (23.0%)
False (correct)	154 (54.4%)
Not sure	64 (22.6%)
To administer an intramuscular injection, pinch the skin and insert the needle at a 45° angle.	
True	35 (12.4%)
False (correct)	211 (74.5%)
Not sure	37 (13.1%)
Apart from the usual symptoms of anaphylaxis, requests for water and thirst shortly after vaccination can hint that anaphylaxis is under way.	
True (correct)	39 (13.8%)
False	89 (31.4%)
Not sure	155 (54.8%)
The quadrivalent influenza vaccine covers against one influenza A H1N1 virus, one influenza A H7N9 virus and two influenza B viruses.	
True	84 (29.7%)
False (correct)	68 (24.0%)
Not sure	131 (46.3%)
For the hepatitis B vaccine, an interruption in the vaccination schedule (missed dose) does not call for extra doses or restarting the entire series of vaccination.	
True (correct)	114 (40.3%)
False	91 (32.2%)
Not sure	78 (27.5%)
The recommended vaccinations for patients <65 years old with chronic liver disease include the influenza vaccine and both pneumococcal vaccines (PCV13 and PPSV23).	
True	138 (48.8%)
False (correct)	90 (31.8%)
Not sure	55 (19.4%)
Perceived knowledge	
Confidence in educating adult patients	
Very Confident	7 (2.5%)
Confident	48 (17.0%)
Moderately Confident	81 (28.6%)
Slightly Confident	95 (33.5%)
Not Confident	52 (18.4%)
Confidence in recommending vaccinations for adult patients	
Very Confident	8 (2.8%)
Confident	55 (19.5%)
Moderately Confident	82 (29.0%)
Slightly Confident	87 (30.7%)
Not Confident	51 (18.0%)
Confidence in administering vaccinations to adult patients	
Very Confident	5 (1.8%)
Confident	6 (2.1%)
Moderately Confident	13 (4.6%)
Slightly Confident	22 (7.8%)
Not Confident	237 (83.7%)
**Attitude and Perception**	
I believe that Pharmacists have an important role to play in providing vaccination services.	
Strongly Agree	81 (28.6%)
Agree	130 (45.9%)
Neither Agree nor Disagree	59 (20.9%)
Disagree	12 (4.2%)
Strongly Disagree	1 (0.4%)
If given the opportunity, I would like to be able to provide direct administration of vaccines to patients.	
Strongly Agree	33 (11.7%)
Agree	67 (23.7%)
Neither Agree nor Disagree	85 (30.0%)
Disagree	70 (24.7%)
Strongly Disagree	28 (9.9%)
Frequency of educating adult patients	
Always	11 (3.9%)
Often	23 (8.1%)
Sometimes	57 (20.1%)
Rarely	112 (39.6%)
Never	80 (28.3%)
Frequency of recommending vaccinations for adult patients	
Always	19 (6.7%)
Often	32 (11.3%)
Sometimes	55 (19.4%)
Rarely	99 (35.0%)
Never	78 (27.6%)
Vaccinations that should be administered by pharmacists (n = 283)	
Influenza	245 (86.6%)
Pneomococcal	149 (52.7%)
Potential COVID-19 vaccine	102 (36.0%)
Hepatitis B	77 (27.2%)
Varicella	61 (21.6%)
Zoster	54 (19.1%)
TdAP	49 (17.3%)
Tetanus Post exposure	42 (14.8%)
Hepatitis A	36 (12.7%)
Measles-Mumps-Rubella	34 (12.0%)
Haemophilus Influenza Type B	27 (9.5%)
Polio	25 (8.8%)
Typhoid	24 (8.5%)
Yellow Fever	23 (8.1%)
Rabies	15 (5.3%)
Cholera	13 (4.6%)
Japanese Encephalitis	1 (0.4%)
Others—2 Human Papillomavirus, 1 Dengue	3 (1.1%)
**Barriers**	
Uncomfortable with injections	198 (70.0%)
Ranked 1st	103 (36.4%)
Ranked 2nd	51 (18.0%)
Ranked 3rd	44 (15.5%)
Lack of knowledge/skills	200 (70.7%)
Ranked 1st	79 (27.9%)
Ranked 2nd	78 (27.6%)
Ranked 3rd	43 (15.2%)
Lack of time	120 (42.4%)
Ranked 1st	40 (14.1%)
Ranked 2nd	30 (10.6%)
Ranked 3rd	50 (17.7%)
Lack of space	83 (29.3%)
Ranked 1st	18 (6.4%)
Ranked 2nd	32 (11.3%)
Ranked 3rd	33 (11.7%)
Lack of support from patients	110 (38.9%)
Ranked 1st	17 (6.0%)
Ranked 2nd	51 (18.0%)
Ranked 3rd	42 (14.8%)
Lack of support from other HCP	101 (35.7%)
Ranked 1st	14 (4.9%)
Ranked 2nd	32 (11.3%)
Ranked 3rd	55 (19.4%)
Others	16 (5.7%)
Ranked 1st	5 (1.8%)
Ranked 2nd	2 (0.7%)
Ranked 3rd	9 (3.2%)
Ranked 1st (n = 283)	
Uncomfortable with injections	109 (38.5%)
Lack of knowledge/skills	80 (28.3%)
Lack of time	41 (14.5%)
Lack of space	18 (6.4%)
Lack of support from patients	17 (6.0%)
Lack of support from other HCP	13 (4.6%)
Others	5 (1.8%)
**Educational Needs**	
Interest in educational programmes	
Yes	247 (87.3%)
No	36 (12.7%)
Areas of interest (n = 247)	
Indication and contraindication	219 (88.7%)
Dose of vaccine, number and freq of doses	207 (83.8%)
Administration techniques	194 (78.5%)
Management of SE and adverse reactions	184 (74.5%)
Patient counselling	180 (72.9%)
Others (sieved)	4 (1.6%)
Type of programme (n = 247)	
Large group lecture/talks	89 (36.0%)
Small group seminar	87 (35.2%)
Hands-on workshop	172 (69.6%)
Web-lecture (webinar)	163 (66.0%)
Modules	168 (68.0%)
Reading materials	138 (55.9%)
